# Concurrent Acute Coronary Syndrome and Pulmonary Embolism in a Patient With Gastric Malignancy: A Diagnostic and Therapeutic Challenge

**DOI:** 10.7759/cureus.107534

**Published:** 2026-04-22

**Authors:** Claire Turnbull, Hazem Elshenawy

**Affiliations:** 1 Internal Medicine, Manchester Royal Infirmary, Manchester, GBR; 2 Cardiology, Manchester Royal Infirmary, Manchester, GBR

**Keywords:** acute coronary syndrome, antithrombotic therapy, cancer-associated thrombosis, gastric neuroendocrine tumour, multidisciplinary management, percutaneous coronary intervention, pulmonary embolism, triple therapy

## Abstract

Acute coronary syndrome (ACS) and pulmonary embolism (PE) are common, potentially life-threatening causes of acute chest pain with significant clinical overlap. Their concurrent occurrence is rare and presents important diagnostic and therapeutic challenges, particularly in patients with underlying malignancy.

We report a case of a male in his 50s who presented with a two-week history of exertional chest pain and dyspnoea. Initial symptoms were attributed to iron deficiency anaemia, and subsequent outpatient investigation identified a type 1 gastric neuroendocrine tumour. He later re-presented with persistent symptoms, and further evaluation revealed bilateral pulmonary emboli and significant coronary artery disease (CAD).

Management was particularly challenging given the competing risks of thrombosis and haemorrhage in the setting of active gastrointestinal malignancy. Following a multidisciplinary team discussion, percutaneous coronary intervention (PCI) was deferred until after tumour resection to mitigate bleeding risk. Following PCI, an individualised antithrombotic regimen was implemented to balance early stent protection with longer-term bleeding risk.

This case demonstrates the challenges of diagnosing and managing concurrent ACS and PE in patients with gastric malignancy, highlighting the need for an individualised, multidisciplinary approach.

## Introduction

Chest pain accounts for approximately 5%-10% of emergency department presentations in the United Kingdom [1]. Acute coronary syndrome (ACS) and pulmonary embolism (PE) are two common and potentially life-threatening causes that require prompt recognition and management. Although they arise from distinct pathophysiological processes, both conditions may present with similar clinical features, including chest pain, dyspnoea, electrocardiographic changes, and troponin elevation.

Although ACS and PE are frequently encountered individually, their concurrent occurrence is rare, with only a limited number of cases reported. Their coexistence presents both diagnostic and therapeutic challenges, as overlapping features may delay recognition, while management is further complicated by the need for concurrent anticoagulation and antiplatelet therapy, substantially increasing bleeding risk [2,3].

Malignancy is a well-recognised prothrombotic state associated with an increased risk of both venous thromboembolism and arterial thrombosis [4,5]. Patients with active cancer are therefore at heightened risk of developing both PE and coronary artery disease (CAD). Current cardio-oncology guidelines provide clear recommendations on the management of PE and CAD individually in patients with cancer; however, they offer limited specific guidance on their concurrent management [6]. This reflects both the rarity of their coexistence and the absence of robust evidence to inform combined management.

We present a case of concurrent PE and significant CAD in a patient with a newly diagnosed gastric neuroendocrine tumour, highlighting diagnostic challenges and the need for an individualised, multidisciplinary approach to management.

## Case presentation

A male in his 50s with no significant past medical history presented with a two-week history of exertional chest pain and dyspnoea. He was clinically obese but had no other cardiovascular risk factors. Physical examination and observations were unremarkable.

Initial electrocardiography (ECG) demonstrated mild T-wave inversion in leads V2-V4, raising concern for possible non-ST elevation myocardial infarction (NSTEMI). Laboratory investigations revealed minimally elevated troponin levels (13 ng/L rising to 15 ng/L) and newly identified iron deficiency anaemia (haemoglobin = 93 g/L, ferritin = 6 µg/L). Repeat ECG showed no dynamic changes; therefore, his chest pain and dyspnoea were attributed to anaemia. He was treated with intravenous iron and referred for outpatient endoscopy, which subsequently identified a type 1 gastric neuroendocrine tumour.

**Figure 1:**
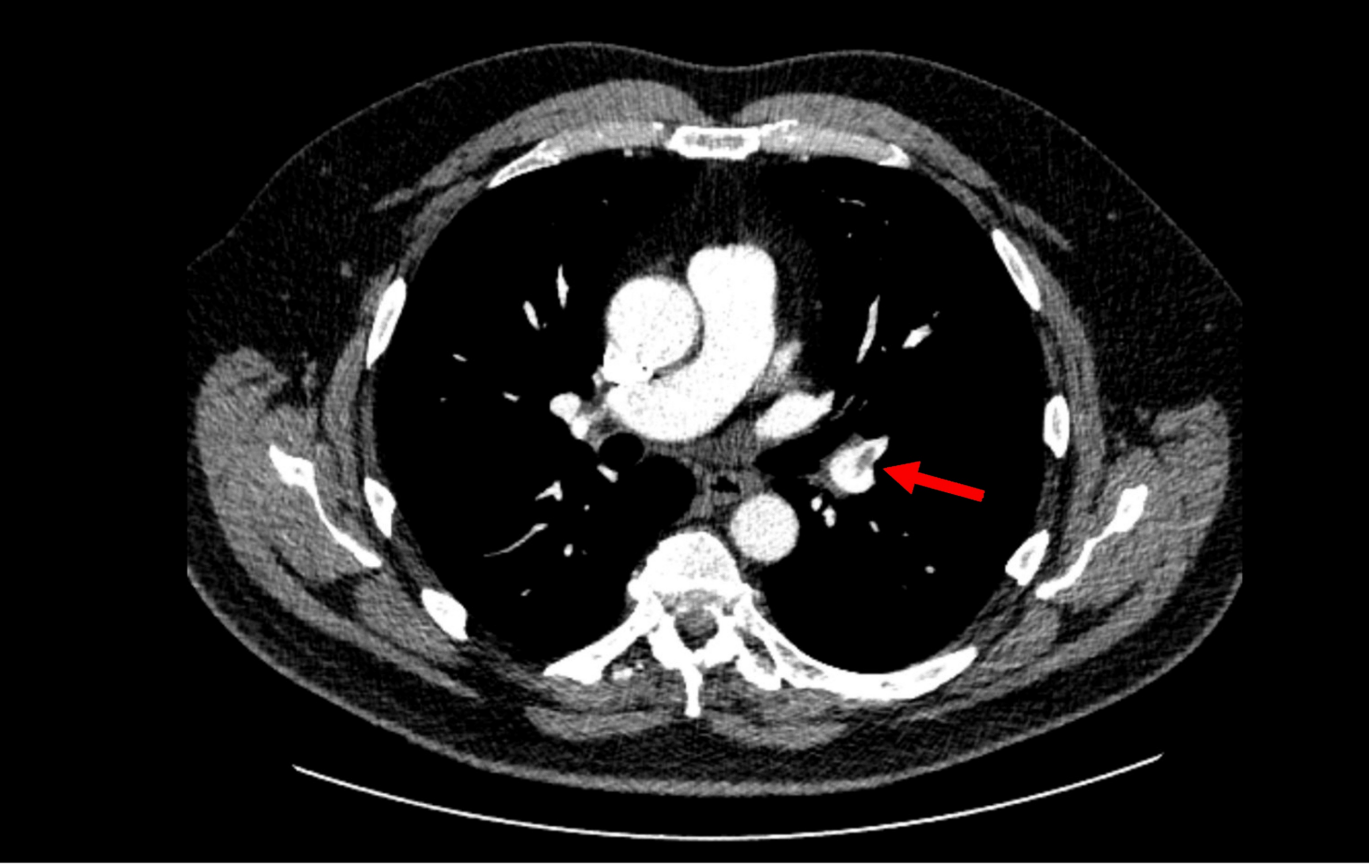
Computed tomography pulmonary angiography demonstrating pulmonary embolus. The red arrow marks filling defects in the left lobar artery suggestive of pulmonary embolism.

Several weeks later, he re-presented with persistent chest tightness and worsening dyspnoea despite iron replacement. D-dimer was elevated (1078 ng/mL), and computed tomography pulmonary angiography (CTPA) demonstrated bilateral pulmonary emboli involving the lobar arteries, as seen in Figure 1. Anticoagulation with rivaroxaban was therefore initiated.

**Figure 2:**
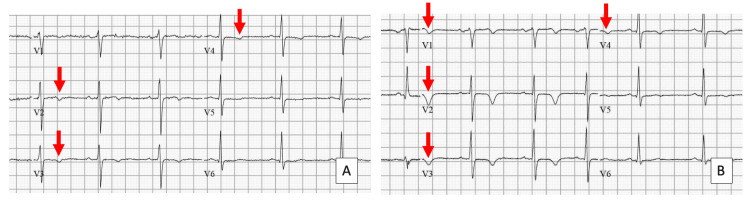
Sequential ECGs demonstrating dynamic changes. ECG at initial presentation (A) shows mild T-wave inversion in leads V2-V4, with progression to deeper T-wave inversion in leads V1-V4 on representation (B).

Repeat ECG demonstrated progression of T-wave inversion in leads V1-V4, as seen in Figure 2, with serial troponin levels elevated but down-trending (25 ng/L to 20 ng/L). A summary of laboratory investigations with accompanying reference ranges can be found in Table 1. These findings were initially attributed to right ventricular strain secondary to PE. However, given persistent symptoms and dynamic ECG changes, the patient was referred to cardiology for further evaluation. Subsequent outpatient coronary angiography demonstrated a mixed atherosclerotic plaque causing 70% stenosis of the proximal left anterior descending artery, consistent with significant CAD.

**Table 1:** Summary of key laboratory investigations. The table summarises diagnostically relevant laboratory investigations from each presentation, alongside reference ranges.

Presentation	Investigation	Result	Reference range (UK)
First presentation	Troponin (initial)	13 ng/L	<14 ng/L
	Troponin (repeat)	15 ng/L	<14 ng/L
	Haemoglobin	93 g/L	130-180 g/L
	Ferritin	6 µg/L	30-400 µg/L
Second presentation	D-dimer	1078 ng/mL	<500 ng/mL
	Troponin (initial)	25 ng/L	<14 ng/L
	Troponin (repeat)	20 ng/L	<14 ng/L

Cardiology recommended percutaneous coronary intervention (PCI). However, the requirement for dual antiplatelet therapy following the procedure, in addition to anticoagulation for PE, posed a high risk of gastrointestinal bleeding in the context of untreated gastric malignancy. Following a multidisciplinary team (MDT) discussion involving cardiology and gastroenterology, a decision was made to defer PCI until after tumour resection.

**Figure 3:**
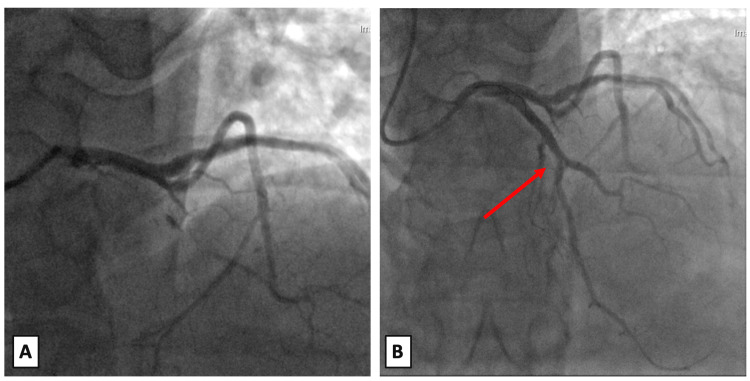
Coronary angiography pre and post PCI. (A) Coronary angiography pre-PCI demonstrates 70% stenosis of LAD. (B) Coronary angiography post-PCI demonstrates successful revascularisation of LAD (as shown by the red arrow). PCI: percutaneous coronary intervention; LAD: left anterior descending artery.

The patient subsequently underwent successful endoscopic mucosal resection of the gastric lesion. This was followed by diagnostic coronary angiography, which demonstrated 70% stenosis of the proximal left anterior descending (LAD) artery lesion. Successful intravascular ultrasound-guided PCI was performed with the placement of two stents, as seen in Figure 3. Following PCI, an individualised antithrombotic strategy was implemented, using a staged approach to optimise early stent protection, while minimising long-term bleeding risk. This individualised approach, in comparison with guideline-directed strategies recommended in other clinical contexts, is summarised in Table 2.

**Table 2:** Post-PCI antithrombotic strategies. The table outlines the post-PCI antithrombotic strategy used in this case, compared with the guideline-directed strategies used in other clinical contexts. PCI: percutaneous coronary intervention; PE: pulmonary embolism; GI: gastrointestinal.

Scenario	Initial phase	Up to 12 months	Long term
This case: PCI with PE and GI malignancy	Aspirin, clopidogrel, and rivaroxaban 10 mg once daily (1 month)	Clopidogrel and rivaroxaban 20 mg once daily (full dose)	Aspirin and rivaroxaban 10 mg once daily
Standard PCI	Aspirin plus a P2Y12 inhibitor (ticagrelor, prasugrel, or clopidogrel)	Dual antiplatelet therapy for 12 months	Aspirin alone
High bleeding risk PCI	Short course dual antiplatelet therapy (1-3 months; usually aspirin and clopidogrel)	Single antiplatelet therapy (usually clopidogrel)	Single antiplatelet therapy
Atrial fibrillation with PCI	Aspirin, clopidogrel, and a direct oral anticoagulant for up to 1 week	Direct oral anticoagulant and clopidogrel	Direct oral anticoagulant alone

Outcome and follow-up

Resection of the gastric neuroendocrine tumour was successful, and the patient remains under gastroenterology follow-up with planned annual endoscopic surveillance. Following PCI, his chest pain and dyspnoea resolved completely, and he has since returned to normal daily activities without limitation. He will remain on aspirin 75 mg once daily and rivaroxaban 10 mg once daily long term for secondary prevention.

## Discussion

Chest pain is among the most common reasons for presentation to the emergency department; however, its broad differential diagnosis can make accurate diagnosis challenging. This is particularly pertinent in cases such as this, where multiple significant pathologies coexist. ACS and PE rarely occur simultaneously, with only a limited number of cases reported in the literature. Despite distinct underlying pathophysiology, ACS and PE often share overlapping clinical features, including chest pain, dyspnoea, ECG changes, and troponin elevation. These overlapping features can present a diagnostic challenge, as findings may be attributed to a single condition, leading to coexisting pathology being overlooked.

In this case, the patient’s initial symptoms were attributed to iron deficiency anaemia, a recognised cause of exertional dyspnoea and chest discomfort. However, current guidelines state that even modestly elevated or dynamic troponin levels, together with ECG changes, should prompt consideration of myocardial ischaemia [2]. Attributing the patient’s symptoms to anaemia, despite mild troponin elevation and T-wave inversion, likely resulted in delayed recognition of underlying CAD.

On re-presentation, troponin levels had increased further; however, interpretation was complicated by the fact that troponin, while sensitive for myocardial injury, is not specific for ACS and may also be elevated in PE [2,3]. In this context, troponin elevation was attributed to right ventricular strain secondary to bilateral pulmonary emboli, reducing suspicion for concurrent myocardial ischaemia and further delaying diagnosis of CAD. This highlights the importance of reassessing patients with persistent or evolving symptoms to avoid missing concurrent pathology.

The main management challenge was balancing the risks of stent thrombosis, recurrent venous thromboembolism, and gastrointestinal bleeding. Following MDT discussion, PCI was deferred until after tumour resection to reduce bleeding risk before starting intensive antithrombotic therapy. This approach is supported by cardio-oncology guidance [6]. Although this carried a potential short-term risk of myocardial ischaemia, it was considered acceptable given the patient’s stable condition and allowed definitive treatment of the tumour. An alternative strategy of immediate PCI with prolonged triple therapy was considered, but deemed to carry an unacceptably high risk of gastrointestinal bleeding, due to gastric mucosal tumour involvement.

Optimising the antithrombotic regimen also required careful consideration. Current guidelines address CAD and venous thromboembolism separately, recommending dual antiplatelet therapy after PCI and therapeutic anticoagulation for venous thromboembolism, but provide limited guidance on their combined use in patients with active malignancy [6-9]. An individualised approach was therefore adopted to balance competing thrombotic and bleeding risks. A short course of triple therapy was used initially, with early transition to dual therapy to reduce bleeding risk, in line with current evidence [8-11]. Dual therapy with clopidogrel and rivaroxaban was then continued, with clopidogrel selected due to its lower bleeding risk [8]. Reduced-dose rivaroxaban was used initially to limit bleeding risk while maintaining anticoagulant effect. This approach is supported by trials such as PIONEER AF-PCI, RE-DUAL PCI, and AUGUSTUS [9-11], although it is important to note these data are extrapolated from atrial fibrillation populations as opposed to patients being treated for PE. As bleeding risk decreased, therapy was simplified to aspirin and low-dose rivaroxaban for long-term secondary prevention.

Only a small number of reports describe concurrent ACS and PE, often identifying prothrombotic risk factors such as malignancy and thrombophilia [12-14]. However, evidence regarding the underlying mechanisms and risk remains limited, as most data are derived from case reports. Notably, this patient had few conventional cardiovascular risk factors aside from male sex and obesity, with no known smoking history, diabetes, hypertension, or dyslipidaemia. This supports emerging evidence that malignancy itself may act as an independent prothrombotic and pro-inflammatory risk factor for both arterial and venous events, even in patients with otherwise low baseline cardiovascular risk [15]. Although a direct causal relationship cannot be definitively established, clinicians should maintain a higher index of suspicion for concurrent ACS and PE in patients with underlying malignancy. This highlights the complexity of managing concurrent ACS and PE in the context of gastrointestinal malignancy, where pragmatic application of existing guidelines and a multidisciplinary, individualised approach are required to balance thrombotic and bleeding risks.

## Conclusions

Concurrent ACS and PE is an uncommon but clinically significant presentation. This case adds to the limited literature on their coexistence by describing the rare combination of ACS, PE, and gastrointestinal malignancy. In keeping with previous reports, it illustrates how overlapping clinical features may delay diagnosis and highlights the importance of considering coexisting pathology in patients with persistent or evolving symptoms.

What distinguishes this case from previously reported examples is the concurrent presence of active gastrointestinal malignancy, which substantially increased bleeding risk in a patient requiring both anticoagulant and antiplatelet therapy. It therefore adds to the literature by illustrating the therapeutic complexity that arises when thrombotic and haemorrhagic risks coexist, and by emphasising the importance of multidisciplinary management when coordinating PCI with tumour resection. It also provides a practical example of an individualised antithrombotic strategy in a clinical scenario where standard guideline-based recommendations may not be directly applicable. Until more robust evidence is available, management of similar cases should remain individualised and guided by multidisciplinary assessment of thrombotic and bleeding risk.
